# Clinical utility of digital pain drawings captured by people living with musculoskeletal pain conditions: a qualitative study

**DOI:** 10.1177/20494637251343227

**Published:** 2025-05-16

**Authors:** Syed Mustafa Ali, Salma Elsayed, Rebecca R Lee, Jill Firth, David McCarthy, William G Dixon, Sabine N van der Veer

**Affiliations:** 1 Centre for Health Informatics, Division of Informatics, Imaging and Data Science, Manchester Academic Health Science Centre, 5292 The University of Manchester, Manchester, UK; 2 National Institute for Health and Care Research (NIHR) Applied Research Collaboration – Greater Manchester (ARC-GM), UK; 3 Centre for Epidemiology Versus Arthritis, Division of Musculoskeletal and Dermatological Sciences, Manchester Academic Health Science Centre, 5292 The University of Manchester, Manchester, UK; 4 NIHR Manchester Biomedical Research Centre, 5293 Manchester University NHS Foundation Trust, Manchester, UK; 5 Integrated Care Centre, Pennine Musculoskeletal Partnership, Oldham, UK; 6 5293 Manchester University NHS Foundation Trust, Manchester, UK; 7 Rheumatology Department, Northern Care Alliance NHS Foundation Trust, Salford, UK

**Keywords:** Clinical decision support, digital pain drawings, manikins, musculoskeletal pain, pain management, pain measurement, patient-generated health data

## Abstract

**Background:**

Digital pain drawings are an emerging method for pain assessment, but it is still unclear how these could best support pain treatment and management decisions. Therefore, this study explored the potential clinical utility of digital pain drawings.

**Methods:**

We conducted a narrative study, involving qualitative interviews with healthcare professionals providing pain management services to people living with musculoskeletal pain conditions working across different disciplines and care levels in the healthcare system of the United Kingdom. We transcribed interviews, conducted thematic content analysis to identify themes and presented results using a framework approach.

**Results:**

We interviewed three general practitioners, five rheumatology healthcare professionals, four physiotherapists, two pain consultants and one rheumatology nurse. We identified four themes describing current pain assessment practices, potential advantages of digital pain drawings either alone or in combination with other pain information (e.g. perceived pain triggers and relieving factors) and outcome measures (e.g. quality of sleep, function and anxiety). Digital pain drawings provide an opportunity of enriching patient-provider communication, particularly for people with language barriers. Digital pain drawings may also support healthcare professionals across different disciplines and care levels (e.g. primary and secondary care) in decisions related to referrals, differential diagnosis, treatment planning, evaluating response to treatment and scheduling follow-up visits when combining pain drawings with other pain information, such as pain consequences and perceived causes.

**Conclusion:**

Digital pain drawings are clinically useful because of their potential to guide diagnosis, treatment and management choices in managing musculoskeletal chronic pain. Future research should investigate how these potential benefits are achieved by integrating digital pain drawings in clinical practice across different disciplines and care levels in the UK’s healthcare system and beyond.

## Background

Chronic pain poses severe socioeconomic and health burden on individuals, healthcare system and societies across the world.^1,2^ Similar to the global trends, the prevalence of chronic pain has been increasing in the United Kingdom, which is estimated to be affecting 28 million adults.^3^ In addition to this increasing prevalence, the management of chronic pain can, at times, be sub-optimal. To improve this, pain management programmes involving multidisciplinary teams have been introduced in the UK.^4^ However, the gap persists between capacity and demands for these services.^5^ Currently, chronic pain is also managed outside the multidisciplinary team settings by different healthcare professionals across primary and secondary levels within the UK’s healthcare system.

Chronic pain assessment is critically important for decision making and developing (and following) treatment plans. However, differences in pain assessment approaches may translate into unwarranted variations in treatment plans and outcomes.^6^ Better pain treatment outcomes are likely to be achieved with improved methods of pain assessment by capturing spatiotemporal aspects of pain, which are recommended by IMMPACT (Initiative on Methods, Measurement, and Pain Assessment in Clinical Trials)^7^ for evaluating pain management interventions and are also needed to develop person-centred treatment plans.^8^ Pain self-reports are considered ‘the gold standard’ for pain assessment^9^ and are preferred by patients in digital formats than paper-based versions.^10,11^ While the existing measures of pain self-report are largely paper-based and do not capture spatiotemporal aspects of pain, digital pain drawings are emerging as an accurate and convenient way^12^ to capture spatiotemporal aspects of pain.^13^ Digital pain drawings are feasible for frequent and timely collection with higher engagement from those who normally are digitally less engaged or excluded (e.g. older adults)^14^ and for automated calculation of summary measures, such as pain extent and pain distribution.^15^

In previous early-stage development studies, healthcare professionals belonging to single disciplines have found potential benefits of digital pain drawings by improving patient-provider communication and aiding clinical decision making.^16–18^ Pain extent and distribution are commonly derived pain information^19,20,21^ and have shown diagnostic and clinical value.^21,22^ However, it is still unclear which aspects of digital pain drawings, either alone or in combination with other outcome measures, has a clinical utility.^23^ Similarly, the Faculty of Pain Medicine has recognised the need to know that how healthcare professionals belonging to different disciplines may prioritise different aspects of pain reports.^24^

Therefore, the aim of this study was to explore perspectives of healthcare professionals from different disciplines involved in pain management on the potential clinical utility of digital pain drawings. This will improve our understanding of how best digital pain drawing can be summarised for supporting clinical decision-making and managing pain effectively.

## Methods

### Design

This is a narrative study,^25^ involving qualitative interviews of multidisciplinary healthcare professionals providing care to people living with musculoskeletal pain conditions. We have reported the findings of this study by following the COREQ checklist^26^ (Annexure 1).

### Ethic statement

As per the institutional guidance, ethical approval was not required for this study because healthcare professionals were asked about clinical judgement and decision-making, which was within their normal clinical responsibilities and remit and without a risk of disclosing their professional conduct.

### Study participants and recruitment

We invited healthcare professionals who provided primary or secondary healthcare services in the UK to adults with musculoskeletal pain conditions. This included rheumatology healthcare professionals (including consultants, specialist registrars and nurses), pain consultants, general practitioners and physiotherapists. Using convenience sampling and snowballing approaches, we identified potential participants through our and participants’ professional networks and invited them by email to partake in the study. Some of these invited professionals were aware of our work on digital pain self-reporting tools prior to their interviews. We included brief information about the study in the email and gave potential participants the opportunity to ask questions about the study and express their interest to participate. Online interviews were scheduled at a mutually convenient time for the researcher and participants. Prior to seeking verbal consent, we provided information about development of an example digital pain manikin,^27^ its feasibility for pain self-reporting,^14^ and its potential use for guiding self-management practices.^28^

### Data collection

**Figure 1.**
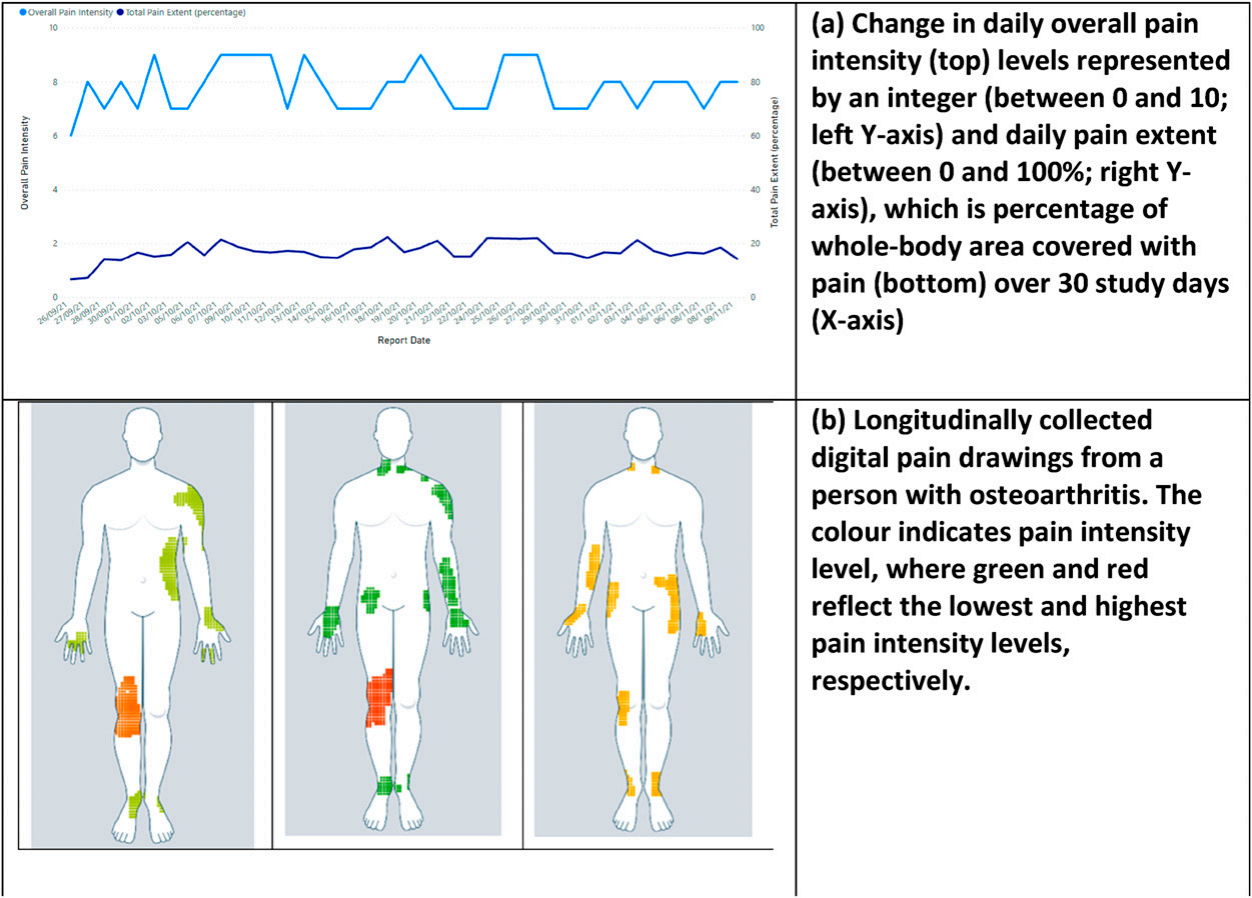
Examples of visual prompts used during interviews. Note: Figures (a) and (b) are taken from our unpublished data collected in our previous study.^14^

We conducted semi-structured interviews via Zoom. We developed an interview guide (Annexure 2) based on previous pain drawing studies,^12,21,29,30^ with topics covering current use of pain assessment tools, how digital pain manikin reports could support delivery of care, impact of their use during clinic consultations and potential barriers to their successful implementation. The topic guide was iteratively refined it over the course of the study in response to preliminary analyses of interview transcripts. We also used visual interview prompts, including digital pain drawings and developed graphs (Figure 1) using data from one of our previous studies^14^ and from systematic reviews on digital pain manikins.^19,31^ We presented five visual prompts to all interviewees and allowed them to share their views on visual(s) of their own choice; some of these visuals related to our previous work on digital pain self-reporting tools. Two researchers interviewed participants together: one male researcher (SMA) had a public health and health informatics background, and one (SE) was a 3^rd^ year medical female student. Data collection and coding of transcripts were done simultaneously and data collection continued until data saturation was achieved. Interviews were audio-recorded and transcribed verbatim by a professional transcription service.

### Analysis approach

We conducted a thematic content analysis at a semantic level and identified themes inductively based on explicit meanings of healthcare professionals’ experiences, that is, without interpreting or presenting underlying meaning. SMA and SE coded transcripts in duplicate, discussed discrepancies and agreed upon a coding structure, which was applied to all transcript by SMA for ensuring consistency. Themes were then derived from a coding structure. Guided by the framework approach, we identified similarities and differences in healthcare professionals’ views^32^ working across different disciplines on the potential of using digital pain drawings in their clinical practice and decision-making processes. All data was imported, processed and analysed within NVivo version 12.^33^

## Findings

Fourteen healthcare professionals were interviewed, including three general practitioners (participants GP1–3), five rheumatology healthcare professionals (i.e. rheumatology consultants, rheumatology registrars and a rheumatology nurse) (Rhe1–5), four physiotherapists (Phys1–4) and two pain consultants (PC1 and PC2). Their years of pain management experience ranged from 3 to 35 years, with average of 11 years (±9 years). Physiotherapists and pain consultants reported some but inconsistent use of paper-based pain drawings in their practice prior to taking part in the interview. Interviews took between 25 and 50 minutes. We identified four themes: (a) current pain assessment practice; (b) perceived advantages of using digital pain drawings in clinic; (c) types of clinical decisions that digital pain drawings could support and (d) additional information to put digital pain drawings in perspective. We describe these themes in more detail below and illustrate them with participants’ quotes.

### (a) Current pain assessment practice

Formal pain assessments, including the use of pain drawings, were inconsistently used across disciplines and care levels. However, none of the healthcare professionals reported to have had any experience of using digital pain drawings as part of their practice. Many routinely asked patients verbally to rate their pain on a scale of 0 to 10. One of the rheumatology consultants described how answering a simple rating question can already be challenging for patients to answer:

‘I don’t use any pain assessment tools beyond discussion with the patient, so I will ask the patients to describe their pain …the location, the intensity. I regularly do use pain scores of, you know, how bad is that out of ten? I think some people sometimes find that difficult to quantify, partly because it changes through time, partly because it’s just a hard thing to do, and partly because there are different locations of your pain and where are you talking about? We do try and break it up in that way but I don’t use any specific pain measurement instruments or tools to clarify that’. [Rhe2 – 13 years of pain management experience]

Specialised pain management services such as pain clinics and physiotherapy asked patients to complete a variety of paper-based questionnaires, including pain drawings, prior to clinical appointments. However, processing and patient non-completion of these questionnaires were highlighted as problems, as described below:

‘The main problems we have with the paper questionnaires…are actually logistical problems. So, for example, the patient receives the questionnaire, they have to return it within two weeks, sometimes they get lost in the post, or sometimes, patients find it difficult to complete the questionnaires, if they find it difficult to read or write, or if they are elderly, for example, and aren't able to complete all the answers, sometimes questionnaires get lost in the post... sometimes, there are problems where questionnaires are not scanned, or not all the pages are scanned [onto the electronic health record] , or sometimes, we've seen the incorrect patients being scanned onto the record’. [PC2 – 5 years of pain management experience]

### (b) Perceived advantages of using digital pain drawings

Healthcare professional described that it was challenging for patients to communicate their experience of pain because of their ability to recall and describe changes in pain correctly and having enough time to do so. However, they thought digital pain drawings could easily convey useful information about pain, especially with the benefit of being collected in real time and in advance of appointments. Participants said:

It [pain drawing] would obviously show to themselves [patients] or to a clinician what, or where and what intensity their pain would be. [Phys3 – 25 years of pain management experience]

So I think…by using colours, it’s quite a good way to show the severity. And generally, you know, it’s very comprehensive. So it asks all the questions that you would normally ask in an initial, and saves time with the assessment as well. [Phys2 – 2 years of pain management experience]

Participants also mentioned that use of pain terminologies was challenging for patients, which was more challenging for people of non-native English backgrounds. Participants mentioned how longitudinally collected digital pain drawings could help overcome these communication challenges, as graphics or visuals were considered a universal language and easier to interpret:

‘Well...it would help them [non-English speakers] more than those who speak English as their native language because it’s visual, isn’t it, and it doesn’t rely on language and misinterpretation of words’. [GP3 – 15 years of pain management experience]

‘They would be able to describe their pain easily and in a detailed way for us, because we know what the certain terminology means and certain ways that they are recording the pain, what colours mean…so they wouldn’t really have to find the words, to explain how severe their pain is’. [Phys – 2 years of pain management experience]

Participants also suggested how services of interpreters for non-English speaking patients could be supported or replaced. One of the participants described it as the below:

‘I think for people where language is a barrier, it would be really helpful…one of the issues, especially if I think about [name of place] when I’ve worked there, the chronic pain consultations were very long and difficult and we didn’t often get an interpreter’. [Rhe4 – 7 years of pain management experience]

Interviewees also described how longitudinally collected digital pain drawings and their summaries could enrich the patient-provider communication by providing a helpful start of their conversation and supporting patients with recalling their pain experiences as described below:

‘I think it can certainly be a starting point...it can enrich the conversation, as the patient who documents sites of pain and ensure they are all addressed and not have to remember that…and if the patient’s also got it on their phone at the same time in clinic, for example, then they can say, doctor, I’ve also got this pain in my back, or my left knee…stuff that wouldn’t otherwise come out’. [Rhe4 – 7 years of pain management experience]

Instead of basing clinical decisions on ‘*snapshots’* and ‘*not having to rely on patients’ recall’*, as described by a physiotherapist, longitudinally collected digital pain drawings could facilitate patient education and shared decision-making by discussing a person’s treatment history in a more collaborative way. One of the rheumatology consultants said:

‘They have treatments that you’ve had longitudinally and you can show… You know, you can say to the patient, well, look, where on this do you think you were bad? Oh, back there in February. Do you think that’s related to medication? Look, we can see that, in February, you had that bad disease, we started the drugs and you can see very clearly that you got better…You’ve got a point of reference that is the patient’s own data’. [Rhe2 – 13 years of pain management experience]

A pain consultant described the scope of patient education and shared decision-making as follows:

‘the purpose of the consultation is mainly to focus on explaining chronic pain to the patient, obviously excluding things like red flags, but also then orientating them towards a rehabilitation approach…there is a lot of educational work that tends to happen within that consultation framework as well. It [digital pain drawing] could [be used] to contextualise some of the discussions with the patient, [for example] with the variability over time, that's a good visual aid to help patients understand fluctuations in pain intensity and how pain is not fixed’. [PC2 – 5 years of pain management experience]

### (c) Types of clinical decisions that digital pain drawings could support

Digital pain drawings alone or in combination with other pain information (e.g. pain triggers) could support a variety of clinical decisions, including referrals (e.g. to rheumatology, physiotherapy and specialised pain management services), supporting consideration of differential diagnoses, evaluating response to treatment, shared treatment planning and tailoring follow-up visits. Table 1 outline the types of clinical decisions and their relevance to different practice areas as mentioned in interviews (supported by visual prompts) along with some illustrative quotes.

**Table 1.** Types of clinical decisions that digital pain drawings could support (theme c).

Type of clinical decision	GP	Rhe	Phys	PC	Illustrative quotes^a^
Referral to secondary and tertiary care settings	X	X	X		‘*if you get to the stage where you feel like you’ve exhausted all your options, nothing really seems to be working, patient didn’t seem to be getting any better. I guess it might help with a referral to the pain clinic*’ [GP1 – 9 years of pain management experience]
					‘*But then if somebody shows up, actually, you know what, the knee is settling down a lot, the colour is changing, the intensity is going down, but it looks like maybe some of these other areas are flaring up or becoming more of a problem. Okay, you’ve got a diagnosis of OA [osteoarthritis], but could there be anything else going on here? Do we need to get this in to see a rheumatologist, do some bloods, check that there isn’t anything maybe systemic? So it could identify other health problems that maybe were missed initially because it was just the OA knee that was on the referral*’. [Phys3 – 25 years of pain management experience]
Differential diagnosis of pain conditions		X		X	‘*It’s, sort of, scattered. It’s affecting all four limbs and the spine. It looks like a characteristic pattern of fibromyalgia so it’s helpful for me around the diagnosis because of the distribution*’. [Rhe2 – 13 years of pain management experience]
					‘*Get more information about the pain…it’s telling me that yes, pain is radiating down to the leg, if it’s a dull pain or a sharp pain, and it will further help me delineating my differential*’ [Rhe3 – 6 years of pain management experience]
					‘*I immediately think about…our patients whom have been given a provisional diagnosis of a radicular back pain or sciatica, is immediately we’re then thinking about dermatomal distribution*’. [PC1 – 9 years of pain management experience]
(Shared) treatment planning	X	X	X	X	‘*Let’s say a patient presents with low back pain, and they’ve had a scan and there’s a disc pressing on the L5 nerve root, and they’ve been told that. But then they draw on their pain map, and there’s no pain radiating all the way down to the foot, then you think, is that true radiculopathy. And then it’s helpful to then use that diagram to show to a patient that actually, I don’t think you would benefit from an intervention or surgery, because it doesn’t seem like the pain you describe is consistent with the problem that you might be trying to solve with surgery’*. [PC2 – 5 years of pain management experience]
					‘*In osteoarthritis, if a patient is saying, I have pain in my hand but my knee is worse so I’ll know which to start off first, which thing to take first, and that’s it, really’*. [GP2 – 3 years of pain management experience]
					‘*That might help me work out […] how to tailor their treatment. Maybe their knee pain is a lot worse than their other joints, mild elsewhere, really severe in their knee*. *I’d give them an intra-articular injection maybe other than an intra-muscular injection’*. [Rhe2 – 13 years of pain management experience]
					‘*With the body map…you looked at the back and you said well, this is where somebody’s more often reporting pain, central sort of lumbar spine, but it doesn’t mean they’re not reporting it in other areas. And actually, the intensity of the pain in the other area, let’s say the shoulder, might be far worse, but it was only there for 1 day. Whereas lumbar spine might have been there every day, but only at a much lower level. And I guess the bit that is important to us is, how much does that impact on the patient? Because if somebody says, I’ve had back pain for 20 years, and I’ve learned to live with it, you know, it’s not why I’m here…it’s just this really big pain that comes in my shoulder once in a blue moon, that’s the thing that bothers me, then that’s clinically what we’re to need to do’*. [Phys3 – 25 years of pain management experience]
Evaluating response to treatment	X	X	X	X	‘*if you’re giving someone a pain relief treatment for the first time, trying to understand whether it’s effective or not is a huge black hole’*. [Rhe1 – 8 years of pain management experience]
					‘*I can see how the severity has changed through time. I might then mentally try and correlate that with other things such as treatments that have been given or external factors that might have exacerbated the disease. Patients, I am sure, would try and do that themselves for the self-management purposes’*. [Rhe2 – 13 years of pain management experience]
					‘*I’ve got a few patients that have got chronic conditions and chronic pain, and they’ll come in every few months asking me to change their medications because they’re not working and they want to try something else. Often, you go back through what they’ve had in the past…so you’ll see patients have already tried amitriptyline, gabapentin, pregabalin, codeine, paracetamol…they’ve gone through them all’.* [GP1 – 9 years of pain management experience]
					‘*They [clinicians involved in treating pain] can maybe use the same tool [digital pain drawings] to continue monitoring response to see whether whatever they’re doing is working better’.* [GP1 – 9 years of pain management experience]
Tailoring timing, frequency and duration of follow-up visits	X		X		‘*I think that [digital pain drawings] will probably save time a lot so I would say it will be very time-effective for a doctor or a clinician because in just one picture, you will decide, okay, I’m going to see this in 5 minutes just to see if there’s tenderness or guarding to decide whether this patient is going to hospital or not’*. [GP2 – 3 years of pain management experience]
					*‘If, say, the data was sent into a consultant to track them or track responding to drugs or something, it might trigger an earlier review. And it might mean that you can cut down on some follow-up slots as well, if patients can actually track their own symptoms and then alert the clinician when they’re potentially going into a flare’*. [Phys1 – 35 years of pain management experience]

Abbreviations: GP = general practitioner; Rhe = rheumatology healthcare professionals; Phys = physiotherapist; PC = pain consultant.

^a^We did not include quotes from all healthcare professional types if these reflected similar views on clinical utility for a particular decision.

### (d) Additional information to put digital pain drawings in perspective

It was consistently reported by participants that correlating different pain aspects (e.g. intensity, location or distribution as depicted in individual pain drawings) or a numerical or graphical summary of digital pain drawings alongside other outcome measures (e.g. quality of life and functions) would help their decision making. Participants suggested aspects of pain (e.g. location, intensity, quality and radiation), pain associated factors (e.g. perceived pain triggers and relieving factors) and other outcome measures (e.g. quality of sleep, work productivity, mood, anxiety, functioning and fatigue) which would enhance the utility of pain drawings:

I mean, as a visual aid itself…I think it's a useful tool, but you would never use that alone to inform any decision-making process anyways…so yes, …you would need some way of asking those questions about exacerbators, what things relieve the pain, how do your levels of distress correlate with that intensity…and functioning levels [PC2 – 5 years of pain management experience]

‘…familiar with SOCRATES [a structured framework of general medical history taking used by many healthcare professionals to assess pain] so I actually still use that. I find it quite useful...I suppose they’re not about severity but I think they do provide very useful information: site, onset, character, radiation, associated symptoms, exacerbating or relieving factors and severity. That’s SOCRATES’. [GP1 – 9 years of pain management experience]

I think it’s important to know everything else about the participants, so core to their sleep function…a few things like, is it impacting your work? Is it impacting your sleep? Is it impacting your mood? Also if a patient gets to score all of this before they come in, it gives a more thorough assessment of things [Rhe4 – 7 years of pain management experience]

Participants also found variations in pain aspects or numeric pain scores useful for their clinical decision-making; correlating them with other outcome measures could support development of personalised treatment plans and advice:

So, I guess from our perspective, with the first graph [referring to visual prompt a in Figure 1] you know, the spread, on the line graph rather than the body chart itself, the interesting thing from our perspective with what one would be looking at, especially on the peaks of the intensity, how that related to maybe what they were doing at the time. So often we use activity diaries for our patients and we use it to help with planning and pacing and prioritising their activities. If we can see, and commonly with patients they tell us they did a particular activity…. And we get them to rate their pain and their fatigue on those days. [Phys3 - 25 years of pain management experience]

…if they can describe whether it’s a dull pain, a sharp pain. Does it happen after certain activities? Does it ease by exercise or does exercise make it worse, or is it the duration of the exercise. Is there a limit? Sometimes we tell patients they have to pace themselves, you know, it’s all quite personalised. [Rhe5 – 8 years of pain management experience]

I can see how the severity has changed through time [referring to a visual prompt 1a]. I might then mentally try and correlate that with other things such as treatments that have been given or external factors that might have exacerbated the disease. [Rhe2 – 13 years of pain management experience]

## Discussion

This narrative study has identified several ways in which digital pain drawings, collected directly from people living with musculoskeletal pain conditions, could support clinical decision making of healthcare professionals belonging to different disciplines involved in pain management. Pain questionnaires were used less regularly in non-specialised pain services (i.e. general practice and rheumatology) and collected more regularly and systematically in specialised services (e.g. physiotherapy and pain clinics) but with processing and utilisation issues. Healthcare professionals thought longitudinally collected digital pain drawings had potential to enrich patient-provider communication, particularly for patients with language barriers, by describing changes in their pain experience, which in turn could support patient education. In addition to enriching patient-provider communication, digital pain drawings could also support a range of clinical decisions (such as referrals, differential diagnosis, shared treatment planning and evaluating treatment response), either alone or in combination with other outcome measures (e.g. physical activity and quality of sleep).

### Relation to other studies

The current pain assessment methods are predominantly paper-based, while digital methods are preferred among both patients and healthcare professionals. We found that ease of communicating pain information, particularly for people with language barriers, was a potential advantage of using digital pain drawings, which is also supported by other studies.^17,18,34^ Though in these studies they interviewed only a few healthcare professionals (e.g. two cancer physicians by Jaatun et al., and one general practitioner and one rheumatologist by Jang et al.), our study involved fourteen healthcare professionals belonging to different disciplines. This helped us understanding how a single aspect of pain may be used by healthcare professionals differently. For example, pain location may help a GP for referring patients, a rheumatologist to diagnose a musculoskeletal condition and a physiotherapist to plan treatment (i.e. pain location impacting function the most will be treated first). We also found that how enriching patient-provider communication may translate into better decisions, as Jang et al. found that healthcare professionals may feel confident about their judgement and decision making.^17^ With potential of enriching patient-provider communication, patients recommend digital pain drawings acquired through handheld devices (e.g. smartphone and tablet), but Spyridonis et al. argued against it for acquiring pain drawings from disabled people.^18^

We also found that digital pain drawings along with other outcome measures are needed for better clinical decisions. The lack of complete pain information may activate providers’ own judgement of patients’ pain experience, hence causing variation in pain management practices.^35^ Capturing, visualising and understanding pain variations and its associated factors are important for clinical decision making^36,37^ as well as for patient education^38,39^ as also mentioned by pain consultants and physiotherapists in our study and in another related study.^34^

### Strengths and limitations

Interviewing healthcare professionals belonging to a variety of disciplines was a strength of this study, because it enabled us developing insights about how they might interpret and use digital pain drawings. However, not involving psychologists was a limitation of this study because pain and anxiety symptoms commonly co-exist. Interviewing psychologists would have provided an additional professional perspective on the potential clinical utility of digital pain drawings by providing insights into how they would corelate pain drawings with mental health related outcome measures. Also, focus group discussions could allow generating further insights into specific aspects of digital pain drawings, which would have a potential to impact clinical decisions across multiple disciplines and care levels. Lastly, interviewing people with lived experience of chronic pain would have added value by providing further insights into some of the patient benefits mentioned by healthcare professionals (e.g. improved pain communication and opportunities to support patient education). However, we have sought patients’ views about digital pain drawings and reported them elsewhere.^28^

### Implications

Pain assessment is a vital step in managing musculoskeletal chronic pain and integrating digital pain drawings (along with other pain information and outcome measures) in clinical practice has a potential to improve both chronic pain assessment and management. However, to harness the full potential of digital pain drawings in clinical settings, they need combining with other pain aspects to support diagnosis and choosing right treatment options. With the feasibility of collecting digital pain drawing from patients and processing them in real-time, digital pain drawing may support differential diagnoses^40^ and reduce delays in diagnosing chronic pain conditions.^41^ Further research should focus on evaluating if integrating digital pain drawings and associated information in clinical practice results into quicker diagnosis and better treatment outcomes.

Digital pain drawings have a potential to improve patient-provider communication, which may also reduce reliance on interpreters for people with language barriers (e.g. ethnic minority groups). As there are pain inequities among ethnic minorities, visualisation of changes in pain can facilitate patient-provider communication and may result in better clinical decisions and treatment outcomes.^28,42^ In addition, future development of digital tools with pain drawing should consider gathering user requirements from other priority groups (e.g. disabled people) to develop more accessible tools. Therefore, future research should focus on exploring how patient-provider communication supported by pain drawings translates into better decision-making and treatment outcomes, and whether this leads to more equitable pain management outcomes for people with diverse backgrounds.

## Conclusion

Digital pain drawings alone or in combination with other pain information and outcome measures are perceived to be potentially useful across disciplines in guiding clinical decisions related to diagnosis, pain treatment and management. Enriching patient-provider communication and resultantly improving clinical decisions are clear potential benefits of using digital pain drawings, particularly for patients with language barriers. Future investigations should focus on understanding how best to integrate pain drawings in care pathways and decision-making processes in a way which improves musculoskeletal pain management and outcomes across different disciplines and care levels in the UK and beyond.

## Supplemental Material

**Supplemental Material -** Clinical utility of digital pain drawings captured by people living with musculoskeletal pain conditions: a qualitative study Supplemental Material for Clinical utility of digital pain drawings captured by people living with musculoskeletal pain conditions: a qualitative study by Syed Mustafa Ali, Salma Elsayed, Rebecca R Lee, Jill Firth, David McCarthy, William G Dixon and Sabine N van der Veer in British Journal of Pain.
