# Supplementary material for: Clinical utility of digital pain drawings captured by people living with musculoskeletal pain conditions: a qualitative study
Source: Br J Pain. 2025 May 16;19(5):326–36. doi: 10.1177/20494637251343227 (PMC12084221; doi:10.1177/20494637251343227)
Supplement: Supplemental Material - Clinical utility of digital pain drawings captured by people living with musculoskeletal pain conditions: a qualitative study [file sj-pdf-1-bjp-10.1177_20494637251343227.pdf]

Consolidated criteria for reporting qualitative studies (COREQ): 32-item checklist

**No. Item**	**Guide questions/description**	Reported on Page #
**Domain 1: Research team and reflexivity**		
Personal Characteristics		
1. Inter viewer/facilitator	Which author/s conducted the inter view or focus group?	Page 5
2. Credentials	What were the researcher's credentials? E.g. PhD, MD	Page 5
3. Occupation	What was their occupation at the time of the study?	Page 5
4. Gender	Was the researcher male or female?	Page 5
5. Experience and training	What experience or training did the researcher have?	Page 5
Relationship with participants		
6. Relationship established	Was a relationship established prior to study commencement?	Page 4 & 5
7. Participant knowledge of the interviewer	What did the participants know about the researcher? e.g. personal goals, reasons for doing the research	Page 4 & 5
8. Interviewer characteristics	What characteristics were reported about the inter viewer/facilitator? e.g. Bias, assumptions, reasons and interests in the research topic	Not reported

**Domain 2: study design**		
Theoretical framework		
9. Methodological orientation and Theory	What methodological orientation was stated to underpin the study? e.g. grounded theory, discourse analysis, ethnography, phenomenology, content analysis	Pages 5 & 6
Participant selection		
10. Sampling	How were participants selected? e.g. purposive, convenience, consecutive, snowball	Page 5
11. Method of approach	How were participants approached? e.g. face-to-face, telephone, mail, email	Page 5
12. Sample size	How many participants were in the study?	Page 5
13. Non-participation	How many people refused to participate or dropped out? Reasons?	Not reported
Setting		
14. Setting of data collection	Where was the data collected? e.g. home, clinic, workplace	Page 4 & 5
15. Presence of non-participants	Was anyone else present besides the participants and researchers?	Page 4 & 5
16. Description of sample	What are the important characteristics of the sample? e.g. demographic data, date	Page 6
Data collection		
17. Interview guide	Were questions, prompts, guides provided by the authors? Was it pilot tested?	Page 5
18. Repeat interviews	Were repeat inter views carried out? If yes, how many?	Not reported
19. Audio/visual recording	Did the research use audio or visual recording to collect the data?	Page 5
20. Field notes	Were field notes made during and/or after the inter view or focus group?	Page 5
21. Duration	What was the duration of the inter views or focus group?	Page 6
22. Data saturation	Was data saturation discussed?	Page 5
23. Transcripts returned	Were transcripts returned to participants for comment and/or correction?	Not reported
**Domain 3: analysis and findings**		
Data analysis		
24. Number of data coders	How many data coders coded the data?	Page 4 & 5

25. Description of the coding tree	Did authors provide a description of the coding tree?	Page 4 & 5
26. Derivation of themes	Were themes identified in advance or derived from the data?	Page 4 & 5
27. Software	What software, if applicable, was used to manage the data?	Page 6
28. Participant checking	Did participants provide feedback on the findings?	Not reported
Reporting		
29. Quotations presented	Were participant quotations presented to illustrate the themes/findings? Was each quotation identified? e.g. participant number	Pages 6 -14
30. Data and findings consistent	Was there consistency between the data presented and the findings?	Pages 6 -14
31. Clarity of major themes	Were major themes clearly presented in the findings?	Pages 6 -14
32. Clarity of minor themes	Is there a description of diverse cases or discussion of minor themes?	Pages 6 -14

Topic guide
Exploring clinical utility of the Manchester Digital Pain Manikin

Interviewer: Provide the background to this study

- In 2018, we developed a prototype digital pain manikin, that includes a human shaped figure, on which a person can directly draw to report location and intensity of pain using their own smartphones.
- In 2021, we improved our digital manikin based on the prototype testing and conducted a feasibility study to assess whether it is feasible for people with MSK pain conditions to self-report their pain using the pain manikin once a day, for 30 days. We found median completion level of 23 reports, out of 30 daily reports.
- We also conducted three ethnicity-specific workshops to improve acceptability of our manikin. We engaged with South Asians, Black African and White British people to know their requirements from a pain self-reporting tool
- Now, we want to explore the clinical utility of patient-reported digital pain manikin reports collected from people with musculoskeletal pain conditions in variety of clinical situations and specifically to explore how digital pain manikin reports can improve the delivery of pain management services

Give opportunity to ask any question, else proceed with the verbal consent and interview.

[recording starts now]

Ask if you are happy to proceed with this recorded interview.

Can you please introduce yourself, telling your job title and explaining your role in pain management?

- How long you have been involved in managing people's musculoskeletal pain?
- What information you prepare or see at the time of consultation?
- What information your prepare or input post consultation?

What is your opinion about existing use of pain assessment tools as part of the clinical care?

- Elaborate, the type of assessment tools and their limitations
- If not in use, then explore how does it limits the overall care delivery
- Visual prompt (showing process of drawing pain and app flow): What is your first impression of this tool for routine and frequent pain assessment?

How routine use of pain manikin reports can aid delivery of clinical care?

[Before getting response from the respondent, show visual prompts for (a) showing trend lines for overall pain intensity and extent; (b) series of manikin reports of a participant having multiple MSK conditions; (c) series of manikin reports of a participants having single MSK condition; (d) pain distribution; (e) heat map; (f) pain symmetry]

- Single manikin report vs multiple reports; explore frequency of collection of pain self-reports, which would be clinically useful.
- Think about the situations in which this tool can be useful (e.g., accident or injury; newly diagnosed painful condition; changes in existing pain patterns, caused by a newly diagnosed painful condition; effectiveness of surgical intervention)
- Relevant to the above situations, what information, gathered on the manikin, can be clinically useful and how?
- What additional information (i.e., outcome measures, patient information, medical history etc) should we consider to collect to improve the clinical utility of manikin-derived information
- Explore self-management opportunities

What consequences/impact do you foresee in tracking pain over time and its visualisation during the consultation?

- How it improves pain communication between provider and patient?
- What might be its value for people with language or communication difficulties? (may refer to series of workshops in the feasibility study)
- What and how the same visual information or a summarised pain score can be beneficial for clinicians and for patients
- What do you feel if it may have a negative impact on the communication or on other aspects of the consultation

What are the potential barriers to successful implementation that we should consider at this stage (when the manikin is not yet integrated with clinical system)?

- Technical difficulties, including providers' capacity building; incentive for provider
- Expectation of and from technology developers
- The Trust-level regulations and guidelines; role of administrators in a Trust for such implementation
- Resource needed for the smooth implementation
